# Flexibility of infrapatellar fat pad affecting anterior knee pain 6 months after anterior cruciate ligament reconstruction with hamstring autograft

**DOI:** 10.1038/s41598-020-78406-y

**Published:** 2020-12-07

**Authors:** Takashi Kitagawa, Junsuke Nakase, Yasushi Takata, Kengo Shimozaki, Kazuki Asai, Rikuto Yoshimizu, Mitsuhiro Kimura, Hiroyuki Tsuchiya

**Affiliations:** 1 Department of Physical Therapy, School of Health Sciences, Shinshu University, 3-1-1 Asahi, Matsumoto, Nagano, 390-8621 Japan; 2 Department of Orthopaedic Surgery, Graduate School of Medical Sciences, Kanazawa University, Kanazawa, Japan

**Keywords:** Medical research, Signs and symptoms

## Abstract

This study aimed to identify factors affecting anterior knee pain (AKP) after anterior cruciate ligament reconstruction (ACLR) with hamstring tendon autograft using ultrasonography. Forty-two patients were evaluated by ultrasound, 6 months after ACLR. The thickness of the superficial part of the infrapatellar fat pad was measured, as well as the thickness change ratio between the two angles. Color Doppler evaluated the rate of blood flow in the fat pad. AKP was assessed with the Kujala Scale. The correlations between AKP and age, body mass index, the thickness change ratio, and the grade of increased blood flow were examined. Independent variables showing significant correlations with AKP were used for multiple linear regression analysis. There were significant correlations between AKP and age (r = − 0.68), body mass index (r = − 0.37), the thickness change ratio of the fat pad (r = 0.73) and the grade of increased blood flow (r = − 0.42), respectively. Age and the thickness change ratio of the fat pad affected the AKP score (R^2^ = 0.56). After ACLR, older age and a decrease in the thickness change ratio of the superficial area of the infrapatellar fat pad appear to affect post-operative AKP after 6 months.

## Introduction

Anterior cruciate ligament (ACL) injury is one of the most common knee injuries, often occurring during sports activities. Patients with ACL ruptures often require surgery, with ACL reconstruction (ACLR) becoming the most common surgical procedure to re-stabilize the knee^1^. Anterior knee pain (AKP), typically related to the donor site, is one of the most frequent complications after ACLR. Anterior knee symptoms include mild pain and discomfort during daily activities and sports. Post-operative AKP is present in up to 18.5–50.0% of knees following a bone-patella tendon-bone autograft ACLR^2,3^. Even when hamstring tendon (HT) grafts are used for ACLR instead, the incidence rate of AKP is up to 9.0–30.0%, as reported previously ^2,3^. One study which investigated post-operative factors in patients with AKP following HT harvesting, reported that increased blood flow in the infrapatellar fat pad (IPFP) was independently associated with the presence of anterior knee symptoms (OR 5.0, 95% CI 1.3–19.9)^4^. Another study revealed older age was the only factor that predicted AKP after ACLR, and those with AKP had a higher body mass index (BMI)^5^. However, the main factors affecting AKP after ACLR are still unclear.

The IPFP is considered as a cause of AKP^6^. The IPFP is one of the four fat pads surrounding the knee joint, being an intra-capsular, extrasynovial structure covered posteriorly by the synovial membrane^7^. This tissue is considered to have a biomechanical role in the patellofemoral joint as well as an inflammatory role, by secreting pro-inflammatory or anti-inflammatory cytokines^8^. One review relating to the IPFP has postulated that the morphology of IPFP changes during knee joint movement^9^. One study suggested an appropriate and reliable clinical instrument for measuring the change in anteroposterior distance of the largest fat lobule between two knee flexion angles^10^. Another study demonstrated that the superficial IPFP layer presented greater elasticity than the deeper one^11^. Recently, one study showed that after ACLR, the thickness change ratio of the superficial part of the IPFP during knee flexion was lower in reconstructed knees than in contralateral knees^12^. These findings indicate that the dynamics of the superficial part of the IPFP during knee flexion decreased after ACLR. Another study investigated the relationship between the range of motion of the knee joint and the dynamics of the IPFP in patients after ACLR^13^. These recent insights may help to explain the decrease in dynamics of the IPFP, particularly in the superficial part of the IPFP, which may cause IPFP impingement or change the pressure of the infrapatellar tissue. Thus, it also might affect AKP after ACLR. After identifying the causes of AKP after ACLR, clinicians were better able to manage AKP. This may present benefits for individuals wishing to return to sporting activities. Ultrasound has been shown to be very useful, not only for the diagnosis of pathologies, but also for treatment^14,15^.

The purpose of this study was to identify the factors affecting AKP after ACLR with HT autograft 6 months post-operatively using dynamic evaluation of ultrasonography. The characteristics of patients and findings from ultrasonography were recorded and evaluated. We hypothesized that the thickness change ratio of the superficial part of the IPFP during knee flexion would affect the AKP after ACLR using HT autograft, due to some underlying pathologies^9–12^.

## Methods

### Subjects

Between October 2015 and October 2017, 42 patients (19 men and 23 women) provided informed consent to have their knees evaluated using ultrasonography 6 months after ACLR. As ultrasound examination was performed only 1 day a week, all available cohort patients were not evaluated (39 of 81 patients). There was no selection bias. Our research protocol conformed to the Declaration of Helsinki, and the study was conducted with the approval of the Science and Research Ethics Committee at Kanazawa university. If the subject was 20 years old or younger, parental consent was obtained. Inclusion criteria were patients who had undergone anatomical single-bundle ACLR using an HT autograft, and who had a full range of motion of their knee joint. Exclusion criteria were patients with multiple ligament injuries and bilateral ACL injuries.

### Surgical procedure and post-operative rehabilitation

All reconstructions were performed arthroscopically by one orthopedic surgeon with the surgical technique according to the previous study^10^. After surgery, the patients all followed the same rehabilitation protocol. Patients were permitted to flex their knee joint from 0° to 90° at 4 weeks post-operatively. Patients were permitted full weight bearing at 2–4 weeks post-operatively and given the recommendation to wear a knee brace for 4 months. At 3 months post-operatively, jogging was permitted, with a return to previous sports activities 6–9 months post-operatively.

### Assessment of AKP and characteristics of patients

Patients answered a 13-item self-report questionnaire, known as the Anterior Knee Pain Scale (also known as the Kujala Scale) to measure the severity of symptoms during activities considered to be specifically associated with AKP syndrome^17^. This valid and reliable patient-reported outcome consists of discrete categories related to symptoms and various levels of current knee function, such as weight bearing, running and jumping^18^. Responses are weighted and summed to provide an overall score between 0–100, where 100 represents no disability/pain. Aside from AKP, age, and BMI were recorded. No patients were prescribed any NSAIDs after 6 months postoperatively.

### Ultrasound assessments

**Figure 1:**
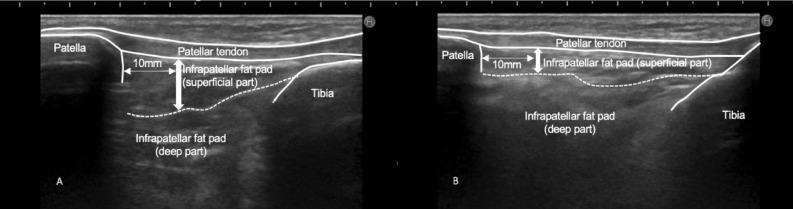
The thickness of the superficial part of the infrapatellar fat pad (IPFP) was measured at a 90° (**A**) and 10° (**B**) knee flexion.

**Figure 2:**
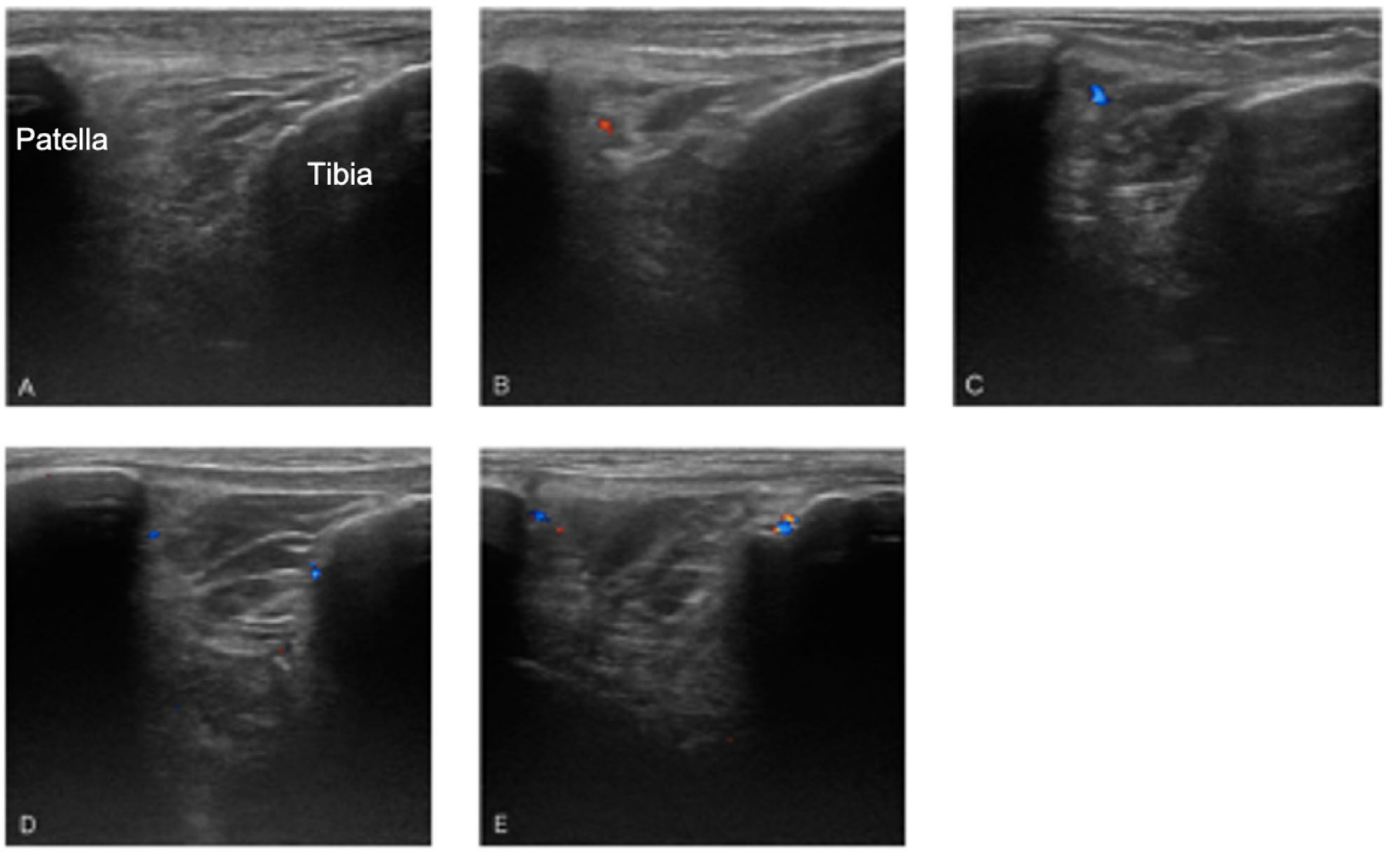
Representative Color Doppler signals. (**A**) shows no blood signals were observed in the IPFP (grade 0). (**B**) and (**C**) Single vessel signal was observed in the IPFP (grade 1). (**D**) and (**E**) More than two vessel signals in less than half of the area of the IPFP (grade 2). Prior to acquisition of data, two examiners examined five randomly selected subjects to assess inter-reproducibility of ultrasound examination. Perfect agreement was observed.

Participants were placed in a sitting position to capture ultrasonography. Ultrasound assessments on reconstructed knees were made with a 5–10-MHz linear transducer (HI VISION Avius, Hitachi Aloka Medical, Tokyo, Japan). As some patients experience difficulty actively extending their knee toward 0°, measurements on reconstructed knees of the thickness of the superficial part of the IPFP was measured at a 10° and 90° knee flexion (Fig. 1). Patients extended their knee from 90° to 10° actively. Given the dynamics of the IPFP during knee movement, the ratio of the change in thickness of the IPFP between the two flexion angles was calculated using the following formula: IPFP thickness change ratio = (the thickness of the superficial part of the IPFP at 90° knee flexion)/(the thickness of the superficial part of the IPFP at 10° knee flexion). A single examiner performed ultrasound imaging of the IPFP using a previously established measurement^12^. Color Doppler was used to detect blood flow in the IPFP according to a previous protocol^4^. The lowest pulse repetition frequency was applied, in order to maximize sensitivity to any flow, by observing the contralateral knee in advance. Where more blood flow signal was noted in comparison with the contralateral knee, this was recorded as increased blood flow. Quantification of the grade of the IPFP blood flow was defined according to a previous study: grade 0: no flow in the IPFP; grade 1: single vessel signals; grade 2: confluent vessel signals in less than half of the whole area of the IPFP (Fig. 2)^19^.

### Statistical analyses

Correlations between the AKP score and age, BMI, IPFP thickness change ratio, and grade of the IPFP blood flow were examined using Spearman's rank-correlation coefficient. Stepwise regression analysis was performed to identify the factors affecting the AKP score. Statistical significance was defined as P < 0.05. For the multiple regression analysis, a power calculation was performed which indicated that a total of 40 patients would be required to show significant difference at an α level of 0.05 and a β level of 80%.

## Results

We examined the factors affecting AKP after ACLR 6 months post-operatively using ultrasonography. Table 1 shows the characteristics and ultrasound measurements of subjects.

**Table 1:** Characteristics of the subjects (mean ± SD).

Characteristic	Value
Age (years)	23.0 ± 10.0
Sex (Male: Female)	19: 23
Height (cm)	165.9 ± 10.6
Weight (kg)	60.4 ± 13.6
BMI (kg/m^2^)	21.7 ± 3.3
The thickness of the superficial part of the IPFP at 90° knee flexion (mm)	10.4 ± 5.0
The thickness of the superficial part of the IPFP at 10° knee flexion (mm)	5.5 ± 3.2
IPFP thickness change ratio (%)	211.0 ± 83.5
The grade of IPFP blood flow (grade 0/1/2)	25/11/6
AKP score	88.8 ± 9.5

*SD* standard deviation, *BMI* body mass index, *IPFP* infrapatellar fat pad, *AKP* anterior knee pain.

**Figure 3:**
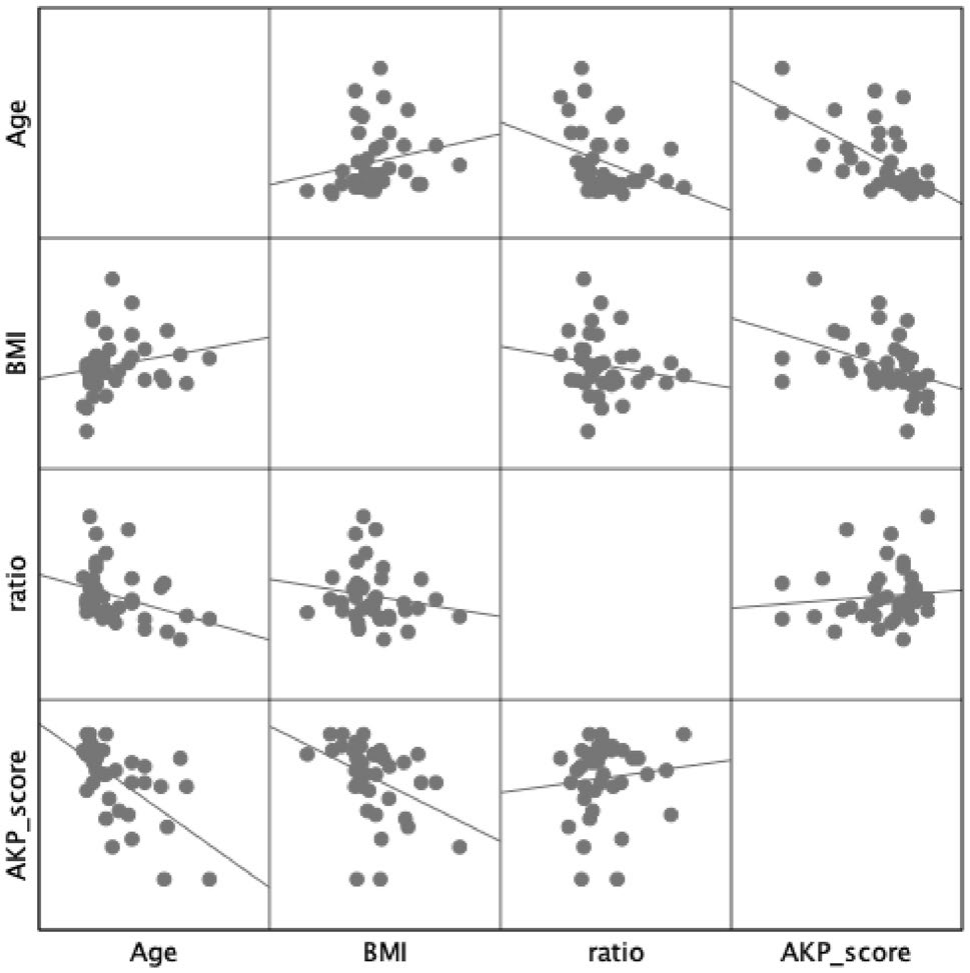
Scatterplots showing the correlation of each variable with regression lines.

Table 2 and Fig. 3 shows the correlation coefficients between the AKP score and other variables. The AKP score showed a significant positive correlation with IPFP thickness change ratio, and significant negative correlations with age, BMI, and grade of the IPFP blood flow. Stepwise regression analysis identified age (standardized coefficient -0.461, P < 0.01) and IPFP thickness change ratio (standardized coefficient 0.417, P < 0.01) as independent variables significantly associated with the AKP score (R^2^ = 0.56) (Table 3).

**Table 2:** Correlation coefficients between characteristics of patients and AKP scores.

	Age	BMI	Ratio	Grade	AKP score
Age	–	0.309*	− 0.493**	0.308*	− 0.683*
BMI		–	− 0.341*	− 0.026	− 0.371*
IPFP thickness change ratio			–	− 0.245	0.728**
Grade				–	− 0.420**
AKP score					–

*AKP* anterior knee pain, *BMI* body mass index, *IPFP* infrapatellar fat pad.

*: P < 0.05, **:P < 0.01.

**Table 3:** Factors associated with AKP scores on stepwise regression analysis.

	Independent variables	Coefficient	Standardized coefficient	t value	P value
AKP score	Age	− 0.438	− 0.461	− 3.872	< 0.01
R^2^ = 0.56	IPFP thickness change ratio	5.690	0.417	3.508	< 0.01

*AKP* anterior knee pain, *IPFP* infrapatellar fat pad.

## Discussion

In this study, we aimed to identify the factors affecting AKP after ACLR with HT autograft 6 months post-operatively using ultrasonography. Results from this study show significant correlations between the AKP score and age, BMI, IPFP thickness change ratio, and grade of the IPFP blood flow, respectively. Additionally, our results report that age and IPFP thickness change ratio were independently associated with the AKP score. ﻿These data suggest that age and IPFP thickness change ratio independently contribute to AKP after ACLR.

A previous study has used the criterion to define post-operative patellofemoral pain of an AKP score less than 87 being considered as having substantial AKP in patients who ﻿had a primary single-bundle ACLR with an HT autograft^5^. According to the criterion administered, our data indicate that there were still some patients who suffered anterior knee symptoms after ACLR with HT autograft 6 months post-operatively. Although the prevalence of AKP has been shown to decrease with time, whichever graft was chosen^3^, it is important to prevent or reduce AKP after 6 months post-operatively, as this is the typical timeframe in which patients return to sporting activities^20^.

As older age was shown to be significantly related to AKP following ACLR, clinicians should be particularly wary of AKP in this population. Moreover, age has also shown to be a predictive factor of insufficient quadriceps strength after ACLR using a hamstring autograft^21^. One other study also reported that knee pain was associated with lower quadriceps strength^22^. Currently, we cannot clarify the exact relationships between age and knee pain after ACLR. Future research should aim to elucidate the mechanism of AKP after ACLR. Specific consideration should also be given to patients with a higher BMI in relation to AKP, as this was identified as one of modifiable factors associated with AKP in the present study. Thus, pre- and post-operative rehabilitation focused on weight control may serve as an effective intervention to prevent the AKP after ACLR.

The quality of the IPFP had been assessed previously using magnetic resonance imaging (MRI). A previous study utilizing second-look arthroscopy has demonstrated that a low intensity area in the IPFP within proton density MRI means there were fibrotic changes in the IPFP post-ACLR^23^. The IPFP is thought to play a biomechanical role that adjusts the pressure of patellofemoral joint and anterior compartment in the knee joint^24^. The fibrosis of the IPFP may cause of AKP, causing higher pressure in the knee joint, and, in turn, decreasing functioning. Moreover, previous animal model studies have demonstrated both histopathological and immunohistochemical changes, including fibrosis, vascularity, and proliferation of small vessels accompanying free nerve endings in the IPFP, after ACLR, trauma, or a patellar tendinopathy model^25–27^. Increased free nerve endings may reduce the threshold of pain sensation. However, further studies focused on restoring or improving the morphology of the IPFP are required.

One of the main findings of this study was that increased blood flow was associated with AKP after ACLR. Abnormal blood flow can be observed in several clinical situations. Some studies have shown that intra-tendinous flow is associated with symptomatic patellar and Achilles tendinopathy, and can serve as a diagnostic indicator of critical situations^27^. However, in our study, multiple regression analysis showed that this is not a risk factor of AKP. Further studies are required to clarify this relationship, using a valid and reliable instrument for the grading of increased blood flow.

The present study has some limitations. Firstly, given our study’s cross-sectional nature, it is difficult to infer causation. However, our study still reveals new information about the pathophysiology of anterior knee symptoms after ACLR with HT autografts, and tested ultrasound as a possible evaluation tool. Second, no pre-operative or functional data were obtained. For better management of AKP after ACLR, future studies should focus on knee instability^28^, and psychological factors^5,29^. Thirdly, given that some data were derived through self-report measurements, this may present accuracy and reliability issues^30^. Lastly, given that we cannot confirm IPFP change ratio and IPFP blood flow as conclusive modifiable factors, this study may not present significant clinical evidence in its current format. Therefore, in future studies we will investigate changes over the course of time, or improvements by way of intervention, using a longitudinal design.

## Conclusion

After ACLR, older age and decrease in thickness change ratio of superficial area of the IPFP affect postoperative AKP after 6 months.
